# Weight and head circumference at birth in function of placental paraben load in Belgium: an ENVIR*ON*AGE birth cohort study

**DOI:** 10.1186/s12940-020-00635-5

**Published:** 2020-07-14

**Authors:** Karen Vrijens, Ilse Van Overmeire, Koen De Cremer, Kristof Y. Neven, Riccardo M. Carollo, Christiane Vleminckx, Joris Van Loco, Tim S. Nawrot

**Affiliations:** 1 Center for Environmental Sciences, Hasselt University, Agoralaan, 3590 Diepenbeek, Belgium; 2 Sciensano, Chemical and physical Health Risks, J. Wytsmanstraat 14, 1050 Brussels, Belgium; 3 Department of Public Health, Environment & Health Unit, Leuven University (KU Leuven), Kapucijnenvoer 35, 3000 Leuven, Belgium

**Keywords:** Paraben exposure, Placental paraben concentrations, Birth cohort, Birth weight and length

## Abstract

**Background:**

Parabens are a group of esters of para-hydroxybenzoic acid utilized as antimicrobial preservatives in many personal care products. Epidemiological studies regarding the adverse effects of parabens on fetuses are limited. The aim of this study was to determine the association between placental paraben exposure and birth outcomes. We assessed paraben concentrations in placental tissue, which potentially gives a better understanding of fetal exposure than the maternal urinary concentrations which are the current golden standard.

**Methods:**

Placental tissue was collected immediately after birth from 142 mother-child pairs from the ENVIR*ON*AGE birth cohort. The placental concentrations of four parabens (methyl (MeP), ethyl (EtP), propyl (PrP), and butyl (BuP)) were determined by ultra-performance liquid chromatography coupled with tandem mass-spectrometry. Generalized linear regression models were used to determine the association between paraben exposure levels and birth outcomes.

**Results:**

The geometric means of placental MeP, EtP, PrP, and BuP were 1.84, 2.16, 1.68 and 0.05 ng/g tissue, respectively. The sum of parabens (∑ parabens, including MeP, EtP and PrP) was negatively associated with birth weight in newborn girls (− 166 g, 95% CI: − 322, − 8.6, *p* = 0.04) after adjustment for a priori selected covariates. The sum of parabens was negatively associated with head circumference (− 0.6 cm, 95% CI: − 1.1, − 0.2, *p* = 0.008) and borderline associated with birth length (− 0.6 cm, 95% CI:-1.3, 0.1, *p* = 0.08). In newborn girls the placental concentration of EtP was negatively associated with head circumference (− 0.6 cm, 95% CI:-1.1, − 0.1, *p* = 0.01) and borderline significantly associated with birth weight and birth length. Lastly, placental EtP and ∑parabens were negatively associated with placental weight in newborn girls but not in newborn boys (− 45.3 g, 95% CI:-86.2, − 4.4, *p* = 0.03).

**Conclusion:**

The negative association between maternal paraben exposure and birth outcomes warrants further research and follow-up over time to determine long term effects of gestational exposure to parabens.

## Introduction

Endocrine disrupting chemicals (EDCs) interfere with the endocrine system, resulting in adverse health effects [1]. It has been estimated that the annual disease costs of EDC exposure exceeds $217 billion in the European Union (1% of the GDP) [2].

Environmental exposure to chemicals that perturb the endocrine balance and inflammatory status are particularly harmful during pregnancy, a time in which an adequate hormonal and oxidative balance, is of utmost importance [3–5]. Parabens, a class of EDCs [6], are esters of p-hydroxybenzoic acid with diverse alkyl substituents and are widely used as preservatives in cosmetics. Although the US Food and Drug Administration (FDA) generally considers them as safe, during the last decade more and more reports have been published on the negative health effects of paraben exposure, including an association with obesity [7], and earlier onset of puberty [8]. The* in utero* period is an extremely sensitive time window for environmental exposures as the fetus develops rapidly and perturbations inferred during this period could have lifelong effects on human health [9]. Recently, researchers started to investigate the potential health impact on the newborn of maternal paraben exposure during pregnancy. As such, maternal urinary paraben concentrations have been associated with an increase in maternal oxidative stress [10], a decrease in mental development in their daughters [11], and a decrease in birth weight [12, 13]. However, until now determination of fetal exposure levels was relying on these maternal urinary measures.

Fetal growth is dependent on genetic, placental, and maternal factors. The fetus is thought to have an inherent growth potential that, under normal circumstances, results in a healthy newborn of appropriate size. The maternal-placental-fetal units act in harmony to provide the needs of the fetus while supporting the physiologic changes of the mother. Low birth weight infants have a 10–20 fold increased risk of dying in the perinatal period [14] and are at increased risk of developing chronic diseases including type 2 diabetes, hypertension and heart disease in later life [15].

Up to now, no reports on placental exposure to parabens and birth outcomes have been reported. Placental concentrations might reflect fetal exposure better than maternal urinary concentrations, as the placenta consists of fetal material and is in close physical contact with the fetus. As such, it could give a clearer picture of fetal exposure.

## Methods

### Study population

We selected 142 participants from the ongoing ENVIR*ON*AGE birth cohort to participate in the current study. These mother-newborn pairs arrived at the hospital East –Limburg for delivery between December 2014 and December 2016. Technicalities of the cohort are described elsewhere in detail [16]. In brief, mothers were asked to participate in the cohort at their arrival in the maternity ward for delivery. The only inclusion criterion being the ability to fill out questionnaires in Dutch. The recruitment protocols have ethical approval from the Ethics Committees of both Hasselt University and East-Limburg Hospital, and are in accordance with the Declaration of Helsinki. Before delivery, all participating mothers provided written informed consent and filled out study questionnaires. Detailed information on maternal age, parity, educational status, occupation, smoking status, alcohol consumption, use of medication, newborn’s ethnicity and paternal age was obtained. Smoking status was classified into three groups: *non-smokers* those who never smoked, *current-smokers* those who continue smoking during pregnancy and *past-smokers* those who quit smoking before pregnancy. Educational status was likewise categorized into three levels: *low-level* without a diploma or only a primary school diploma, *middle-level* with a high school diploma and *high-level* with a college or university diploma. Lastly, the maternal body mass index (BMI), gestational weight gain, method of delivery and data on health complications were provided by the medical records from the hospital archives.

#### Birth outcomes: birth weight, birth length, head circumference and placental weight

Birth weight, length, and head circumference were obtained at birth from hospital maternity records. The date of conception was estimated on the basis of the first day of the mother’s last menstrual period combined with the first ultrasonographic examination, to accurately calculate gestational age of the newborn. Placental weight was determined after birth and prior to taking placental biopsies for molecular and (bio) chemical analyses. For this, after the umbilical cord was removed, placentas were placed on a HR2393 scale (Philips, Amsterdam, Netherlands) and weight was assessed without any handling of the placenta to 1 g precision.

### Placenta paraben concentrations

Placentas were frozen at − 40 °C within 10 min after birth and stored until homogenization. After thawing, the chorio-amniotic membrane was removed before further processing. Approximately half of the placenta (fixed orientation) was homogenized using a Knife mill Grindomix GM 200 (Retsch, Haan, Germany) during two 20 s pulses at 10.000 rpm, in order to minimize site-variation within the placenta. Homogenized samples were frozen at − 20 °C until analysis.

The levels of MeP, EtP, PrP and BuP were assessed using a previously published sensitive method [17]. In brief, a fraction of homogenized placenta (1.5 g) was weighed in a polypropylene tube and 150 μL of internal standard mixture solution was added. Tert-butyl methyl ether/hexane was added and incubated for 10 min, samples were then centrifuged and the organic layer was transferred to a clean tube. Samples were dried and the residue re-dissolved in a 50/50 solution of H_2_O and MeOH, subsequently filtered across a 0.20 μm PVDF filter (Whatman, Diegem, Belgium) prior to UPLC-MS/MS analysis.

UPLC-MS/MS analysis was performed on an Acquity UPLC (Waters, Milford, MA, USA) system in combination with a triple quadrupole XevoTQ-S mass spectrophotometer. The limits of detection (LOD) were defined as the mean concentration in the blank plus 3 times the standard deviation of the blank. LODs were 0.1 (MeP), 0.2 (EtP), 0.1 (PrP) and 0.2 (BuP) ng/g respectively. Quality control was performed using spiked matrices at three different concentration levels. For samples below the LOD, the value was substituted as LOD/2, apart for BuP, as none of the samples were detected above the LOD, the exposure to BuP was dropped from further analyses.

The sum of parabens was calculated as the combined exposure level to MeP, EtP and PrP for each individual.

### Statistical analysis

**Fig. 1:**
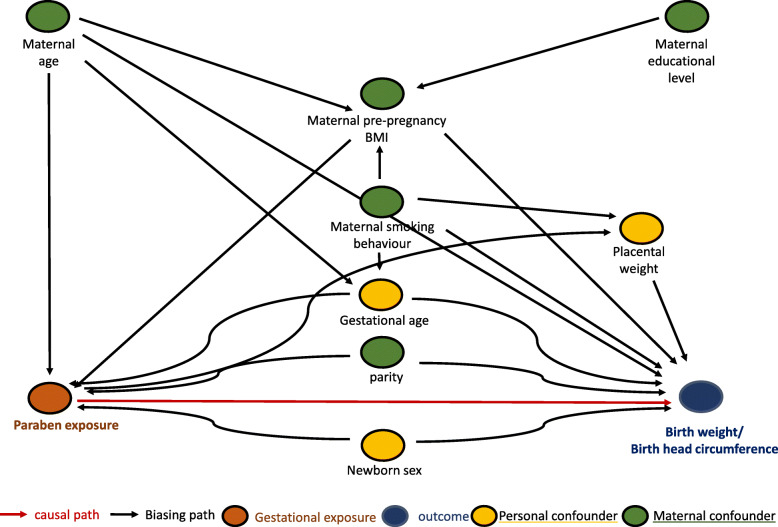
Directed acyclic graph of paraben exposure and growth parameters at birth

All statistical analyses were performed using SAS (Version 9.4, SAS Institute, NC, USA) software. Multivariate linear regression models were used to examine the relationship between placental paraben concentrations and birth outcomes. We constructed a directed acyclic graph (Fig. 1) to guide the selection of covariates using DAGitty [18]. From the DAG, we included maternal age, maternal pre-pregnancy BMI, maternal smoking behavior and educational level, parity, newborn sex, and gestational age as covariates in our models. Due to missing data for smoking behavior (*n* = 1) the final dataset contained 141 mother-newborn pairs for analysis. We additionally stratified our study population by sex and corrected the model for the same confounders as above apart for sex. We calculated the interaction term for sex in the association between paraben exposure and birth outcomes, to better understand the role sex potentially plays in this association between exposure and outcome. The presence of a significant interaction indicates that the effect of the exposure variables on the measured birth outcomes is different for both sexes.

## Results

Detailed information on the study population is shown in Table 1. Participating mothers were on average (SD) 30.3 (4.3) years of age, with an average body mass index (BMI) of 25.2 (5.0). The majority of the mothers (69.5%) never smoked and had a high educational level (51.1%). The newborn in the study population was the first child for 44.6% of the mothers. Their newborn was born after, on average, 39.5 (1.1) weeks with an average birth weight of 3463 (405) g. Approximately half of the newborns were boys (52.5%). Placental weight immediately after delivery averaged 510 (103) g. Data on smoking were missing for 1 individual, resulting in a final study population of 141 mothers.

**Table 1:** Description of the study population characteristics (*n* = 141)

Characteristic	mean (SD)	no.	%
**Mother**			
age (years)	30.3 (4.3)		
pre-gestational body mass index	25.2 (5.0)		
educational level			
Low		16	11.5
Middle		53	37.6
High		72	51.1
Parity			
1		63	44.6
2		56	39.8
3		22	15.6
smoking behaviour			
never smoked		98	69.5
before pregnancy		33	23.4
during pregnancy		10	7.1
**Newborn**			
sex, *male*		74	52.5
gestational age (weeks)	39.5 (1.1)		
birth weight (g)	3463 (405)		
birth length (cm)	50.4 (1.8)		
head circumference (cm)	34.4 (1.3)		
placental weight (g)	510 (103)		

Table 2 shows the geometric mean and the 25th, 75th and 90th percentiles of placental paraben concentrations. Average (SD) levels for MeP were 1.84 (5.73) ng/g, for EtP 2.16 (2.68) ng/g, for PrP 1.68 (14.5) ng/g, and for BuP 0.05 (0.05) ng/g and for the sum of the three parabens MeP, EtP and PrP 4.05 (7.85) ng/g. Only EtP was detected in the majority (88%) of placental samples above the LOD, whereas no samples had BuP concentrations above the LOD. Therefore, BuP concentrations were excluded from the sum of parabens. As we noted major differences between newborns girls and newborn boys in association with exposure, we tested for the interaction between sex and exposure, however this was not significant, *p* = 0.48 for EtP and *p* = 0.38 for ∑parabens.

**Table 2:** Placenta paraben concentrations (ng/g) among 141 mother-newborn pairs from the ENVIR*ON*AGE birth cohort

Analyte	Mean	Std Dev	25th Pctl	75th Pctl	90th Pctl	LOD (ng/g)	% > LOD
**MeP**	1.84	5.73	0.05	0.05	5.75	0.1	19
**EtP**	2.16	2.68	0.62	2.82	4.88	0.2	88
**PrP**	1.68	14.50	0.05	0.05	0.93	0.1	15
**BuP**	0.05	0.05	0.01	0.09	0.13	0.2	0
**∑ parabens**	4.05	7.85	0.70	3.87	10.84		

∑ parabens includes the amounts of MeP, EtP and PrP for each individual from the study population

Table 3 shows the results from the association analyses between placental paraben concentrations and placental weight. The sum of all parabens (∑paraben) was negatively associated with placental weight after adjustment for a priori selected covariates: maternal age, maternal pre-gestational BMI, smoking behavior and educational level, as well as parity, gestational age and sex of the newborn. Both EtP and ∑paraben exposure showed a trend towards a negative association with placental weight (respectively *p* = 0.11 and *p* = 0.08). When stratifying the data by sex of the newborn the negative association between exposure and placental weight became significant in girls, with *p* = 0.02 for EtP exposure and *p* = 0.03 for ∑paraben exposure. The results for boys alone were not significant.

**Table 3:** Associations of placental paraben concentrations and placental weight within the ENVIR*ON*AGE birth cohort (*n* = 141)

	Girls and boys		Girls		Boys	
**Analyte**	**adjusted β (95%CI)**	***p*-value**	**adjusted β (95%CI)**	***p*-value**	**adjusted β (95%CI)**	***p*-value**
**EtP**	−24.8 (−55.5, 6.0)	0.11	−51.7 (−96.1, −7.2)	0.02	−28.4 (−76.4, 19.7)	0.24
**∑ parabens**	−25.2 (−53.5, 3.1)	0.08	−45.3 (−86.2, −4.4)	0.03	−26.4 (−69.9, 16.9)	0.23

Results from adjusted analyses included maternal age, maternal pre-gestational BMI, smoking behavior and educational level, as well as parity, gestational age and sex of the newborn as covariates

Table 4 shows the results from the association analyses between placental paraben concentrations and birth parameters including birth weight, birth length and head circumference. After adjusting for a set of a priori selected covariates (maternal age, maternal pre-gestational BMI, smoking behavior and educational level, as well as parity, gestational age and sex of the newborn, see Fig. 1), placental EtP and ∑paraben concentrations showed a negative association with birth weight, birth length and head circumference.

**Table 4:** Associations of placental paraben concentrations and birth parameters within the ENVIR*ON*AGE birth cohort, *n* = 141

		Girls and boys		Girls		Boys	
**Analyte**	**Parameter**	**adjusted β (95% CI)**	***p*-value**	**adjusted β (95% CI)**	***p*-value**	**Adjusted β (95% CI)**	***p*-value**
**EtP**	birth weight, g	− 101 (− 212.3, 11.1)	0.07	− 165 (− 337, 6.2)	0.06	−61.2 (− 231.1, 108.7)	0.47
	birth length, cm	−0.5 (−1.04, 0.03)	0.06	−0.6 (−1.4, 0.1)	0.08	−0.1 (−1.0, 0.8)	0.77
	head circumference, cm	−0.4 (− 0.8, 0.03)	0.07	− 0.6 (− 1.1, − 0.1)	0.01	−0.3 (− 1.0, 0.4)	0.42
**∑ parabens**	birth weight, g	− 101 (− 203.9, 1.9)	0.05	− 166 (−322, −8.6)	0.04	− 54.0 (− 208.5, 100.5)	0.49
	birth length, cm	− 0.5 (− 0.9, 0.1)	0.07	− 0.6 (− 1.3, 0.1)	0.08	−0.1 (− 1.0, 0.7)	0.72
	head circumference, cm	−0.4 (− 0.7, 0.01)	0.06	− 0.6 (− 1.1, − 0.2)	0.008	−0.3 (− 0.9, 0.4)	0.46

Results from adjusted analyses included maternal age, maternal pre-gestational BMI, smoking behavior and educational level, as well as parity, gestational age and sex of the newborn as covariates

After stratifying by sex, the observed associations appear to be driven by the girls, as they have stronger associations than the total population combined, whereas results in boys alone are not significant. In girls, placental EtP exposure was significantly inversely associated with head circumference (*p* = 0.01) and showed a negative trend for birthweight (*p* = 0.06) and birth length (*p* = 0.08), whereas placental sum of paraben exposure was significantly and inversely associated both with head circumference (*p* = 0.008) and with birth weight (*p* = 0.04) and showed a negative trend for birth length (*p* = 0.08).

## Discussion

We found an inverse association between placental EtP and sum of paraben (EtP, MeP and PrP) exposure and birth weight, birth length and head circumference in newborns from the ENVIR*ON*AGE birth cohort. We furthermore observed a trend for a negative association between placental EtP and sum of paraben exposure and placental weight. Whether the placental paraben exposure levels cause a direct decrease in birth weight, or whether this is mediated through a reduction in placental weight (Fig. 1) remains to be elucidated. It is possible that paraben exposure throughout pregnancy affects the growth of the placenta first, leading to a smaller placenta, which ultimately results in a smaller newborn. It has been reported that placental growth characteristics are more important than placental weight in the determination of birth weight [19] hence it is possible that the effect of placental paraben exposure on birthweight, is partly due to an effect of exposure on placental weight. However, we only measured placental weight and have no information on other placental characteristics such as diameter, thickness and vascularization, which would be necessary to study this in more depth.

Our manuscript is the first to describe placental exposure levels of parabens. To the best of our knowledge, previous studies on the relation between maternal exposure during pregnancy and birth outcomes in the newborn have been based on urinary concentrations. Maternal urinary concentrations might not reflect fetal responses well, as the fetus is more sensitive to the effects of EDC exposure than adults [20]. Furthermore, a study comparing maternal urinary, serum and fetal amniotic fluid for levels of bezophenones [21] showed major differences between the matrices. Between maternal and fetal levels of benzophenones, a group of chemicals commonly used as UV filters, a large difference in concentrations measured between maternal urine (1000 times higher), maternal serum (ten times higher) and amniotic fluid or cord blood as a proxy for fetal exposure was reported. Apart from bezophenone-3 (BP-3), the BP compound with the highest maternal exposure levels, no correlations could be established between maternal and fetal exposure levels [21]. Similarly, a study in rats has shown that levels of EtP and BuP differ significantly between rat amniotic fluid and maternal plasma after subcutaneous administration [22]. These results call for accurate measurement of fetal exposure levels to understand the biological consequences of exposure.

Current literature on maternal paraben exposure during pregnancy in association with birth parameters is inconsistent. In a Taiwanese population of 199 mother-newborn pairs, maternal urinary MeP levels were negatively associated with birth weight, birth length, and head circumference, although only in female newborns [12]. A large Chinese study population including 1006 mother-newborn pairs found no significant associations between maternal urinary paraben concentrations and birth weight. They only observed a modest positive association with birth length, only in boys, and only for the boys in the higher tertiles of exposure [23]. In the PROTECT cohort, based in Puerto Rico, a study on 922 pregnant women showed decreased odds for small for gestational age (SGA) amongst newborns with maternal urinary BuP, PrP, and MeP concentrations [24]. Finally, findings from the French EDEN cohort on 473 mother-son pairs assessing urinary concentrations for 9 phenols (including 4 parabens) and 11 phthalate metabolites identified only significant associations with placental weight, not with birth weight. For placental weight, they noted a positive association with exposure to the sum of parabens [25]. The apparent discrepancy on placental weight with our results could be due to the matrix chosen for exposure assessment, as we used the same set of covariates in our analysis as was done within the EDEN cohort.

The biological mechanisms involved in the observed association between placental paraben levels and birth parameters is unclear. Although it is known that parabens have weak estrogenic activity in vitro [26], it is unclear whether this is relevant in humans at the current exposure levels. Furthermore, parabens are also known to interfere with the thyroid hormones. In vitro studies showed MeP was able to prevent the synthesis of thyroid hormones (TH) by preventing iodide organification [27] and in vivo work in rats demonstrated a weak TH receptor agonist activity for BuP [28]. More recently, a significant inverse association between urinary PrP exposure and total T4 was demonstrated in adults from the NHANES study [12, 29].

Lastly, maternal urinary EtP was negatively associated with interleukin-1β in mothers from the LIFECODES cohort [29], indicating the potential for the inflammatory response to be involved in the biological mechanisms between paraben exposure and birth parameters. However further research is warranted to investigate the underlying biological pathways.

Although our reported findings have relatively large *p*-values, just around or below the standard 0.05 cut-off point of significance, we believe our data are not chance findings based on their associated estimated effect sizes, i.e. sum of placental paraben exposure in newborn girls is associated with a considerable difference in birthweight of 166 g (95% CI: − 322, − 8.6). Furthermore, the investigated variables indicating newborns’ size, including birthweight, birth length and head circumference, show consistent findings between these antropometeric outcomes at birth and paraben exposure. Because we tested an a priori hypothesis involving interrelated outcomes (birth weight, birth length, newborn head circumference) as well as exposure (sum of parabens). Therefore, the newborn birth outcomes or exposure indicators did not provide a completely independent opportunity for a type I error. Hence, we did not perform an approach on multiple comparisons as this considers independence between the different statistical analysis.

Our estimates of in utero paraben exposure and birth outcomes in girls have a public health relevance. Indeed increasing the placental paraben exposure from the 25th to the 75th percentile resulted in an 112 g reduction in birth weight. This is a relevant reduction compared with other exposures, as exposure to second-hand smoke (SHS) in non-smoking women reduced the mean BW of their infants by 53 g, and even with 92 g in actively smoking mothers exposed to SHS compared to active smoking mothers not exposed to SHS [30]. In addition, head circumference has been associated with intelligence [31]. Our study has several strengths. We are the first to assess paraben exposure levels in placental tissue, and associate this with birth outcomes, rather than the more traditional method of using maternal urinary paraben exposure levels. Our findings are generalizable because our study population is representative of the gestational segment of the Flemish population [32]. However, the study also has some potential limitations. The assessment of exposure occurred in placental tissue collected immediately after birth, and although we assume the placenta reflects long term exposure, we only measure exposure once, at the time at birth. Due to a lack of data on parabens’ toxicokinetics within placental tissue and its accumulation over the pregnancy we cannot interpret the timing of exposure within pregnancy with our current data. Furthermore, we have not been able to incorpate the fact that the fetus is usually co-exposed to multiple parabens as well as other EDCs in our analyses.

## Conclusions

In conclusion, our study showed a significant inverse association between placental ethyl paraben exposure and birth weight, birth length and head circumference, both before and after adjustment for selected covariates. We also observed a negative association between the sum of placental paraben exposure and birth weight and birth length, only after adjustment for selected covariates. A trend for a negative association with placental weight was furthermore observed both for EtP and sum of paraben exposure. Further studies are necessary to identify the underlying molecular mechanisms behind these associations.

## Data Availability

The data included in the manuscript is available from the responsible author, Tim S Nawrot, upon request (tim.nawrot@uhasselt.be).

## Abbreviations

BMI: Body mass index
BuP: Butylparaben
CI: Confidende interval
DAG: Directed acyclic graph
EDC: Endocrine disrupting chemical
ENVIR*ON*AGE: Environmental influence on healthy ageing
EtP: Ethylparaben
FDA: Food and drug administration
GDP: Gross domestic product
LOD: Limit of detection
MEP: Methylparaben
PrP: Propylparaben
PVDF: Polyvinylidene difluoride
SHS: Second hand smoke
UPLC-MS/MS: Ultraperformance liquid chromatography-tandem mass spectrometry
